# Unlocking Dreams and Dreamless Sleep: Machine Learning Classification With Optimal EEG Channels

**DOI:** 10.1155/bmri/3585125

**Published:** 2025-02-10

**Authors:** Luis Alfredo Moctezuma, Marta Molinas, Takashi Abe

**Affiliations:** ^1^International Institute for Integrative Sleep Medicine (WPI-IIIS), University of Tsukuba, Tsukuba, Ibaraki, Japan; ^2^Department of Engineering Cybernetics, Norwegian University of Science and Technology, Trondheim, Trøndelag, Norway

**Keywords:** automatic dream detection, channel selection, electroencephalography, feature extraction, machine learning, sleep

## Abstract

Research suggests that dreams play a role in the regulation of emotional processing and memory consolidation; electroencephalography (EEG) is useful for studying them, but manual annotation is time-consuming and prone to bias. This study was aimed at developing an EEG-based machine learning (ML) model to automatically identify dream and dreamless states in sleep. We extracted features from EEG data using common spatial patterns (CSPs) and the discrete wavelet transform (DWT) and used them to classify EEG signals into dream and dreamless states using ML models. To determine the most informative channels for classification, we used the permutation-based channel selection method and the nondominated sorting genetic algorithm II (NSGA-II). We evaluated our proposal using a public dataset that is part of the DREAM project, which was collected from 58 EEG channels during rapid eye movement (REM) and non-REM sleep, while 28 subjects reported dream or dreamless experiences. We achieved accuracies greater than 0.85 to distinguish dream and dreamless states using CSP-based feature extraction combined with *k*-nearest neighbors (KNN), as well as through multiple combinations of EEG channels identified by channel selection methods. Our findings suggest that as few as 8–10 EEG channels may be sufficient for dream recognition. Excluding one subject at a time during model training revealed challenges in generalizing the models to unseen subjects. Channel selection methods have proven to be effective in selecting relevant subsets of EEG channels to classify dreams and dreamless experiences. Our results demonstrate the feasibility of automatic dream detection and highlight the need to improve ML generalization.

## 1. Introduction

During sleep, our brain generates a stream of consciousness composed of vivid and realistic sensory experiences, thoughts, and emotions, which we call dreams. This common human experience has been extensively explored since ancient times; dreams are multifaceted phenomena analyzed from the poetic, philosophical, physiological, and psychological perspectives [1–4].

Although the exact purpose and mechanisms of dreaming remain elusive, scientific research has provided valuable insights into these intriguing experiences. Research indicates that dreams play a crucial role in emotion processing and memory consolidation [3, 5–12]. Evidence suggests that dreams occur during both rapid eye movement (REM) sleep and nonrapid eye movement (NREM: N1, N2, and N3) sleep. When awakened during REM sleep, subjects report having had a dream in 80% of cases, compared to 50% of cases when awakened during NREM sleep. Furthermore, emotionally and perceptually vivid dreams are reported more frequently after awakening from REM sleep [6, 7, 13–19]. Studies using functional magnetic resonance imaging (fMRI) reveal a strong activation in areas associated with emotion, memory, and sensory processing during REM sleep, contributing to the vividness and emotional intensity of dreams [14].

Recalling a dream immediately after awakening requires attention, as it dissipates quickly. Waiting until the morning to record the dream content can result in inaccurate information [1]. For this reason, dream emotions have been extensively studied through self-reports obtained when sleep is interrupted (serial awakening paradigm) [1, 7, 20].

Dream reports can be used to study emotions in dreams, providing insights into the human psyche, symptoms of psychiatric and sleep disorders, and understanding the nature of consciousness. Dream emotions and their alterations, such as recurrent nightmares and lucid dreams, are core symptoms of psychiatric and sleep disorders, including post-traumatic stress disorder (PTSD), nightmare disorder, narcolepsy, and others [1, 12, 21, 22].

Currently, approaches to dream report analysis include self-written reports and data collected by electroencephalography (EEG), electromyography (EMG), electrooculography (EOG), and fMRI [6, 23–27]. To collect dream reports in conjunction with EEG data, subjects must awaken at night to report whether they have dreamed and, in some studies, rate their emotional experience using the Self-Assessment Manikin (SAM) (from 1 to 9, indicating pleasantness/stimulation) or the Positive and Negative Affect Scale (PANAS) (from 1 to 5, indicating happiness/unhappiness) [12, 22, 24, 27–29]. The procedure is tedious and lengthy, requiring the expertise of a sleep specialist to manually label the sleep stages for further analysis.

Studies utilizing fMRI have involved two to three subjects who were awakened to provide verbal reports describing their visual experiences. Keywords related to visual objects or scenes were manually extracted and mapped to WordNet/ImageNet for categorizing the fMRI data. Results obtained using support vector machines (SVMs) and convolutional neural networks (CNNs) indicate that the decoded features could identify the dreamed object category with low accuracy, yet still above chance levels [25, 26].

A study investigated the presence versus absence of dreams during NREM sleep stages using dream reports. Data from 25 EEG channels (EEG power spectrum), along with two EOG and two EMG channels, were recorded from nine subjects during the first 3–4 h of sleep. The authors applied the evidence accumulation clustering (EAC) algorithm to form two clusters: (1) subjects who did not report a dream and (2) subjects who reported a dream experience [7]. The reported accuracy ranged from 0.54 to 0.59, underscoring the complexity of the awakening paradigm and the challenges of analyzing NREM sleep dream reports.

Other approaches to classifying dream emotions using EEG signals include analyzing beta waves and frontal alpha asymmetry (FAA) [22, 23]. In a study that used FAA during REM sleep, the authors of [22] analyzed dream reports, showing that it is possible to identify anger in dreams. In particular, subjects with a higher relative alpha power on the right (F4 channel) compared to the left (F3) in the frontal region experienced more anger in their dreams.

State-of-the-art approaches primarily use the power spectrum and peak alpha frequency for statistical analysis and visual comparison to analyze differences between EEGs with dream and dreamless content, with a recent shift towards automatic classification [24, 27, 29–31].

Due to these visual comparisons and the variability in the EEG data collection protocols, current research is highly inconsistent. For example, dream recall during NREM has been associated with decreased alpha activity [32], while other studies report the opposite [33]. It has also been associated with a decrease in spindle frequency power and delta power [34], but again, some studies report contrary findings [35]. Another inconsistency is the association of dream recall with different brain regions: the frontal region [32], the left frontal and temporoparietal cortex [36], or the posterior parietal/occipital cortex [6, 37]. However, a consistent observation across studies is that reduced power in the delta and/or slow oscillation bands can predict the dream experience [6, 17, 32–36].

Automatically decoding the emotional content of dreams without awakening using a low-density EEG device will dramatically accelerate the process of analyzing dream experiences in both experimental research and clinical settings. Research on dream emotions has begun using machine learning (ML) and deep learning (DL) algorithms; however, it is necessary to continue exploring robust methods with high classification performance that can contribute to the development of real-time models for dream detection and decoding [24, 26, 27].

The subjective and elusive nature of dreams presents a significant challenge for ML-based detection. Individual differences in dream experiences and expressions can confound algorithms trained on limited data. Automatic dream detection is a burgeoning field with immense potential to unlock the secrets of sleep and consciousness. Advances in this area could pave the way for novel human–computer interactions and creative expression tools inspired by the rich imagery and symbolism of dreams.

Previously, we developed ML/DL models using six EEG channels to detect dream emotions during REM sleep based on a public dataset [24]. These models not only characterized the complexity of the problem but also showed promising results, achieving accuracies and area under the receiver operating characteristic curve (AUROC) greater than 80%. This was accomplished using features based on the discrete wavelet transform (DWT) with gradient boosting (GB) or random forest (RF) algorithms, as well as with a CNN called EEGNet [27].

In this study, we aim to advance research in dream detection by developing and evaluating new methods for feature extraction and classification. Our goal is to not only optimize feature extraction and classification techniques for the automatic classification of dreams and dreamless states but also to gain insights into the spatial distribution of dream-related brain activity. To achieve this, we use the nondominated sorting genetic algorithm II (NSGA-II) and permutation-based channel selection to identify the most relevant EEG channels, contributing to more efficient and effective automatic classification of dreams and dreamless states.

## 2. Materials and Methods

### 2.1. EEG Dataset and Preprocessing

Recent efforts have aimed to unify datasets related to dream report research using EEG, EMG, ECG, and EOG signals [28]. Leveraging this initiative, we used a public dataset described in [17], which includes dream and dreamless reports from sleep onset, N2, and REM. In this dataset, subjects were awakened to provide open-ended verbal reports on their mental experiences. EEG epochs containing dreams and dreamless content were evaluated by two raters, achieving a 97.4% agreement rate; disagreements were resolved by a third rater.

The dataset includes EEG data from 28 subjects, recorded with 58 EEG channels from the BrainProducts cap using the 10–10 system, with a sampling rate of 400 Hz [17]. Subjects reported dream experiences 23% of the time, and 77% reported dreamless experiences.

**Figure 1:**
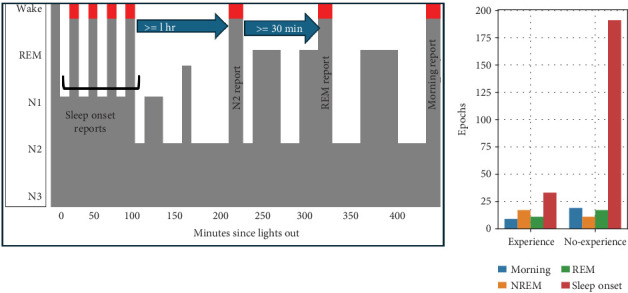
Protocol for data collection and class distribution during the sleep onset, N1, REM, and morning report [17].

During the first hour of the night, up to 10 dream reports of *sleep onset* were collected, which occurred during various stages of NREM: 61% from N1, 38% from N2, and 2% from N3. One hour after the last dream report of “sleep onset,” another report was collected when N2 was visually detected. Subsequently, at least 30 min later, another report was collected when REM sleep was visually detected. The subjects then slept uninterrupted until they were awakened around 8 a.m. to provide a final dream report. The process and distribution of reports across different sleep stages are illustrated in Figure 1.

EEG channels were re-referenced by subtracting the average of the right and left mastoids (*A*1 + *A*2)/2, and the dataset was then downsampled to 200 Hz. To increase the number of instances, each epoch was divided into 2, 5, 10, or 15-s segments. As shown in our previous research, using these segment sizes yielded similar or better performance compared to using the entire epoch [27]. Based on experimental results, we report findings using 2-s segments to maximize the number of EEG instances in the dataset. We also tested various band-pass filter frequencies and found that a range of 0–35 Hz provided the highest performance, so we applied this band-pass filter range. Depending on the experiment configuration, features were extracted from the EEG signal segments and used as input for the ML algorithms, as detailed below. We tested all combinations and selected the best parameters by iteratively creating ML models using 10-fold cross-validation.

### 2.2. Common Spatial Pattern (CSP)–Based Feature Extraction

CSPs and principal component analysis (PCA) are well-established techniques for EEG signal processing. PCA is a technique for dimensionality reduction and noise reduction, applied to EEG data to extract the most informative components. CSP, on the other hand, is used for feature extraction by finding spatial filters that enhance discriminatory information between different tasks. This involves calculating the covariance matrix of the EEG signals and deriving generalized eigenvectors from these matrices to obtain the spatial filters, which are designed to maximize the variance in one class while minimizing it in the other.

The first required step is to separate the dataset into a training set and a test set. In the training set, CSP filters are computed using labeled EEG data for each class. These filters are then used to transform EEG signals into a new set of features that highlight relevant spatial patterns for classification.

We applied CSP to the EEG data to obtain spatial filters that enhance class-specific patterns. Subsequently, PCA was applied to further reduce the dimensionality and capture the most informative components. For both CSP and PCA, the number of components was set to the number of channels in the dataset. After feature extraction, the features were standardized by removing the mean and scaling to unit variance using methods implemented in the scikit-learn Python library [38].

### 2.3. DWT-Based Feature Extraction

DWT effectively analyzes nonstationary signals by offering a time-frequency representation that captures both low and high frequencies. It offers a detailed view of the original signal while significantly reducing the computation time by passing the signal through a series of low-pass and high-pass filter pairs [39].

DWT uses a multiresolution analysis approach to decompose the signal into different scales or resolutions. EEG signals are decomposed into approximation coefficients (representing low-frequency components) and detail coefficients (representing high-frequency components) at each level. This decomposition is applied iteratively to the approximation coefficients, forming a tree-like structure.

In our previous research, we decomposed EEG signals into various sub-bands and extracted features, demonstrating strong performance in EEG-related tasks. Building on these results, in this study, we applied DWT with four levels of decomposition to extract five sub-bands from each EEG channel (four arrays of detail coefficients and one approximation per EEG channel). Based on experimental evidence and previous research findings, we utilized the mother wavelet biorthogonal 2.2 [40, 41].

For each sub-band, we computed 10 features that have demonstrated good performance in previous EEG signal research: Selvik fractal dimension, Katz fractal dimension, Petrosian fractal dimension, Higuchi fractal dimension, instantaneous energy, Teager energy, Hjorth mobility, Hjorth complexity, kurtosis, and skewness [27, 40–42]. This process was repeated for each channel individually, and the resulting features were concatenated into a single feature vector for each instance.

### 2.4. Classifiers

The feature vectors obtained from the CSP- or DWT-based feature extraction processes were input into several well-known ML algorithms for comparison: GB, SVM, *k*-nearest neighbors (KNN), logistic regression (LR), and multilayer perceptron (MLP) [43–47]. Below is a brief description of the methods of interest in our work since, as will be explained later, those methods are the ones with which we obtained the highest performance.

GB focuses progressively on complicated cases, offering an optimal technique for handling unbalanced datasets by enhancing the impact of positive labels. It combines weak learners (e.g., decision trees) to create strong learners capable of accurate predictions using gradient descent to minimize the loss function [43]. GB trees allow us to retrieve importance scores, summarizing which features were most useful for the construction of such decision trees and for the prediction task. GB has demonstrated superior performance over other algorithms, particularly in binary classification problems [43–46].

LR explores the relationship between dependent and independent variables, focusing on how well predictor variables can predict the dependent variable, and examines the conditional probability distribution of the response given the predictor values [48, 49].

SVM offers a global solution, with classification complexity independent of feature dimensions, and is flexible in representing complex functions. SVM uses hyperplanes, defined by support vectors, to separate data classes by maximizing margins [50].

KNN stores training data instances and learns based on the KNN, where *k* is an integer value determined experimentally and highly dependent on the data. The *k* data points most similar to a new data point in the training dataset are localized, and the prediction is obtained by applying the majority vote on the *k*-nearest data points [51, 52].

MLP is a fully connected feedforward neural network that uses backpropagation for training. It consists of an input layer, one or more hidden layers with many neurons stacked together, and an output layer. The inputs are combined with the initial weights in a weighted sum and are subjected to the activation function. Each layer feeds the next with the result of its calculation, looping through all hidden layers to the output layer [53].

All ML methods were implemented in Python using the scikit-learn library [38]. For algorithms in which hyperparameters were available, we selected them experimentally; for example, in the case of KNN, we tested the performance of the models using up to 20 neighbors and automatically selected the best *k* for each experiment. We divided the EEG dataset into 70% for training and 30% for testing, evaluating performance with accuracy, *F*1 score (referred to as *F*score in this document), precision, recall, and AUROC. We used a 10-fold cross-validation for performance evaluation and reported the average results. The results presented correspond to the performance of the test set unless otherwise specified.

### 2.5. Channel Reduction and Selection

While laboratory settings and research-grade EEG equipment provide controlled environments and high-quality multichannel recordings, they may not be suitable for certain applications, situations, or populations. Traditional EEG systems face challenges such as high computational costs, equipment immobility, and the complexity of high-density setups.

The main objectives of channel reduction and selection are to (1) reduce computational costs for EEG signal processing, (2) mitigate overfitting by eliminating unnecessary channels and improving classification performance, as many channels may contain redundant or irrelevant information, (3) identify brain areas generating task-dependent activity, and (4) create power-efficient and easy-to-use portable EEG systems, reducing preparation time. All of these objectives can be achieved by selecting the most relevant channels and removing redundant and task-irrelevant channels [41, 54].

To address these challenges, we used a computationally efficient permutation-based channel selection method along with the multiobjective optimization (MOO) algorithm NSGA-II [40, 41, 55].

#### 2.5.1. Permutation-Based EEG Channel Selection

Permutation-based channel selection effectively identifies the most relevant channels for ML-based sleep stage classification, as demonstrated with models of five and two classes [55, 56]. This method systematically evaluates the impact of each EEG channel on classification performance, providing a data-driven approach to identify the most informative channels.

The process begins by creating a model with the 58 EEG channels available in the dataset, for which it uses 70% of the data for training. The remaining 30% of the data serves as a test set to predict dream or dreamless states and measure performance, establishing the *baseline performance*.

We extracted data from the first EEG channel of all instances in the test set and randomly reassigned it to different instances. This alteration ensures that the EEG channel data no longer corresponds to its original instance. Since the model was trained to associate specific information with this channel position, we can assess the importance of the EEG channel information by observing its impact on the classification performance for the test set instances.

To compute the performance change (performance delta), we calculate the average difference across the metrics: accuracy, *F*score, precision, recall, and AUROC. This procedure is applied to all EEG channels, allowing us to identify which channels contribute to performance increases or decreases in the test set, thus highlighting their importance in classifying EEG signals with dream and dreamless content. In addition, the process is repeated using 10-fold cross-validation to ensure that the important channels identified remain consistent across different test sets.

**Figure 2:**
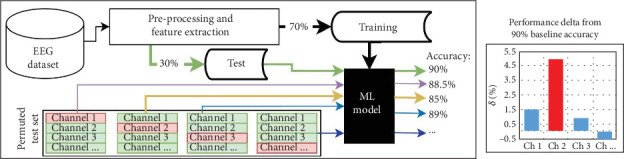
Flowchart illustrating the permutation-based process for channel selection. After the preprocessing and feature extraction process, the ML models are created using 70% of the dataset (training set), and the ML model is used to predict the instances in the test set and obtain the baseline performance (90% in the example). Then, we permute/shuffle the data from one channel at a time (the channel indicated in red in the permuted test sets area), ensuring that the information from all instances is different in the channel position under analysis, and this can obtain a different performance with the modified test sets that indicate whether the permuted/shuffle channel is relevant or not to keep or improve the performance. In the example presented, Channel 2 is the most relevant since, when changing the information from that channel position, the performance decreases by 5%. The comparison showing the performance delta for each permuted/shuffled channel is presented in the subfigure on the right.

Figure 2 illustrates this process, highlighting that *Channel 2* is the most relevant, as its removal results in the greatest performance decrease compared to other channels.

#### 2.5.2. NSGA-Based Channel Selection

A MOO problem involves maximizing or minimizing two or more objective functions by systematically selecting input values from a valid set and evaluating the function values, which may be subject to constraints [57]. NSGA-II is a widely used MOO algorithm that identifies a set of solutions superior to others in the search space when all objectives are considered but potentially inferior for one or more specific objectives. Solutions are deemed feasible if they satisfy all constraints and optimal if they achieve the best values for the objective functions. These solutions are referred to as Pareto front solutions or *nondominated solutions*, while the rest are called dominated solutions. The algorithm employs a nondominated sorting ranking selection method to prioritize promising candidates and uses a niching mechanism to maintain diversity within subpopulations [57–59].

We use NSGA-II to select the most relevant channels for classifying dreams versus dreamless states. This algorithm has successfully identified important EEG channels in various applications with two to five objectives [27, 41]. NSGA-II addressed certain limitations of its predecessor, including high computational complexity, a nonelitist approach, and the need to specify a sharing parameter to maintain diversity within the population. Reduced the computational cost from *O*(*MN*^3^) to *O*(*MN*^2^), where *M* is the number of objectives and *N* is the population size. Furthermore, NSGA-II introduced an elitist mechanism by comparing the current population with the best nondominated solutions identified in previous iterations [59].

**Figure 3:**
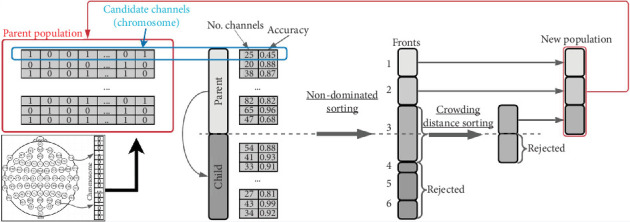
Illustration of NSGA-II process: chromosome representation, population diversity, and objective-based front sorting to generate new populations with the best candidates.

Figure 3 illustrates the NSGA-II process. In this example, each gene represents an EEG channel, and each chromosome in the population represents a combination of these channels. The objectives are to minimize the number of channels while increasing or maintaining accuracy. The algorithm calculates the Pareto fronts based on these objectives to select the best candidates for the next generation. It uses the parent population to generate the offspring (or child) population through genetic operations like crossover and mutation, and the next generation's parent population is then selected from a combined pool of parents and offspring [59].

**Figure 4:**
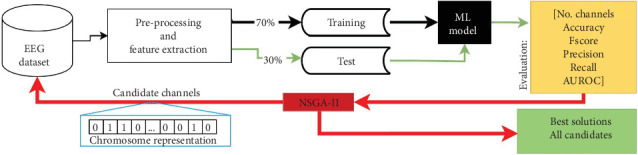
Flowchart of the optimization process for electroencephalographic channel selection using the nondominated sorting genetic algorithm II (NSGA-II). Note: AUROC, area under the receiver operating characteristic curve.

Figure 4 presents the flowchart of the optimization process used in this study. It is defined by six unconstrained objectives based on the NSGA-II structure: (1) decrease the number of EEG channels required to classify EEG signals with dream and dreamless content, while (2–6) increase or at least maintain the classification performance in the five different metrics used to evaluate test sets (accuracy, *F*score, precision, recall, and AUROC). We use a chromosome with binary values to represent the 58 EEG channels, where each gene indicates whether a channel is included in the classification process (1) or not (0).

The process begins by generating chromosomes in the population, which represent an iteration of NSGA-II and are candidates for the best channel configurations. EEG data corresponding to channels marked as 1 in each chromosome is extracted, and then the ML models are trained using 70% of this data and tested in the remaining 30%.

This process is repeated with populations of 15 chromosomes, a value determined experimentally. The optimization process ends based on an objective space tolerance of 0.001, which is evaluated every 10 generations. If the optimization goals are not met, the process stops after a maximum of 200 generations, also determined experimentally.

## 3. Results

### 3.1. Exploring the Effectiveness of CSP-Based and DWT-Based Feature Extraction for Dream and Dreamless Classification Using EEG Signals

**Figure 5:**
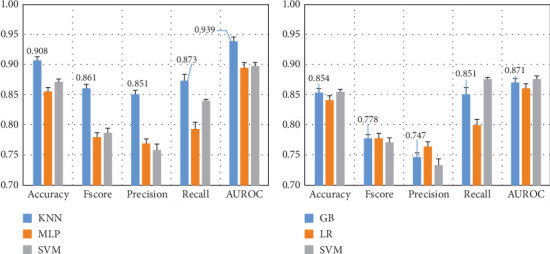
Dream and dreamless classification from electroencephalographic signals using features based on (a) common spatial patterns and (b) discrete wavelet transform, both with the top three machine learning algorithms using information from 58 electroencephalographic channels, including the instances from the full dataset. Note: KNN, *k*-nearest neighbors; MLP, multilayer perceptron; SVM, support vector machine; GB, gradient boosting; LR, logistic regression; AUROC, area under the receiver operating characteristic curve.

Here, we compare the two methods for feature extraction implemented in this study: (1) CSP-based feature extraction and (2) DWT-based feature extraction to decompose EEG signals into different sub-bands and compute 10 features per sub-band. After preprocessing the dataset and extracting the features of each EEG channel, we feed them into various ML algorithms.  Figure 5 presents the results for the Top 3 algorithms for both feature extraction methods.

Figure 5 demonstrates that the CSP features combined with KNN yield the highest performance, with a 10-fold cross-validation standard deviation of approximately 1%, indicating model robustness and consistency. DWT features result in a 5% performance drop across most metrics, with *F*score and precision decreasing by 10%. Thus, subsequent experiments focus on CSP with the KNN algorithm.

### 3.2. Impact of Awake Assumption and Dataset Balancing on EEG-Based Dream Classification

In describing the original datasets, the authors comment that when some of the dream reports were collected, the subjects were probably awake. This could occur in the sleep onset or in the morning reports, as the time between one report and the next was random. However, reports collected after visually confirming N2 or REM sleep stages were free of this assumption.

**Figure 6:**
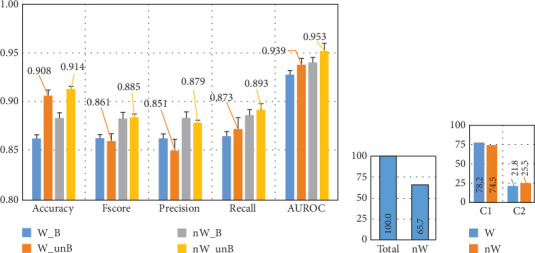
Dream and dreamless classification using common spatial patterns for feature extraction and KNN. (a) Comparison of the classification performance of dream versus dreamless experience from electroencephalographic signals using different sub-dataset configurations: W_B includes instances where subjects were likely awake and balancing the dataset, W_unB includes awake instances without balancing the dataset, nW_B does not include the awake instances and the dataset is balanced, and nW_unB do not include awake instances, and the sub-dataset is unbalanced. (b) Nonawake instances compared to the total. (c) Distribution of the instances, considering W or nW instances. Note: AUROC, area under the receiver operating characteristic curve; C1, dreamless; C2, dream.

To assess the impact of the awake assumption on model performance, we conducted a set of experiments with and without these dream reports. As shown in Figure 6(b), when considering only dream reports with awake assumption not reported in the public dataset, 34.3% of the dataset is removed, which could reduce the performance due to the low number of instances from which the model can learn.  Figure 6(c) shows the distribution of the subsets when awake is assumed or not, which illustrates that instances are present in a similar way in both classes.

**Figure 7:**
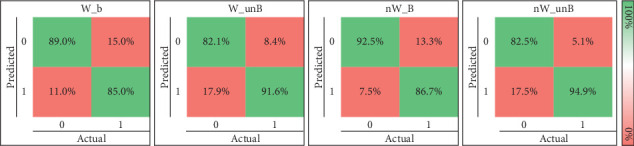
Confusion matrices of the four different experiments presented in Figure 6, which use features based on common spatial patterns for dream versus dreamless classification from electroencephalographic signals. Note: 0 indicates dreamless class and 1 indicates dream.

We also evaluated the effect of dataset balancing. Balancing the subdatasets refers to the use of the same number of instances as the class with fewer instances, which were randomly chosen in each fold from the class with more instances. The results obtained are presented in Figure 6(a). W_B shows the performance using only instances with the awake assumption of the authors of the dataset and balancing the dataset, and W_unB without balancing the subdataset. nW_B present indicates the performance considering only instances where there is no awake assumption and a balanced dataset, and nW_unB without balancing the subdataset. The confusion matrices for the four different experiments are presented in Figure 7.

The results indicate that unbalanced datasets consistently lead to better performance, regardless of whether dream reports with the awake assumption were included. When comparing these results with the analysis of the entire dataset in Figures 5 and 6(a), the performance difference is minimal, averaging less than 0.02.

Figure 6(a) also shows that when the subdatasets are balanced (using the same number of instances for both classes as the class with fewer instances), the classification of dreamless reports decreases by approximately 7% and 10%, respectively, for both, when including instances with the awake assumption or not. On the other hand, the correct classification of dream reports increases by 6% and 8%, respectively. In addition to the segregation of the instances with the awake assumption, this may also be due to the smaller number of instances used to train and test the ML models.

Based on these findings, we chose to proceed with the full dataset (unbalanced, including reports with the awake assumption) for further analysis of dream and dreamless EEG signals.

### 3.3. Assessing ML Generalizability on Dream and Dreamless Classification From Unseen Subjects Using a Leave-One-Subject-Out Approach

In this experiment, we aimed to evaluate the generalizability of ML models for detecting dream and dreamless states in unseen subjects using a leave-one-subject-out of the training set approach. Once the ML model is trained with the data from 27 subjects, the EEG data from the excluded subject is used for testing the performance to classify the dream reports. This process was repeated for each of the 28 subjects, and the average performance was reported.

**Figure 8:**
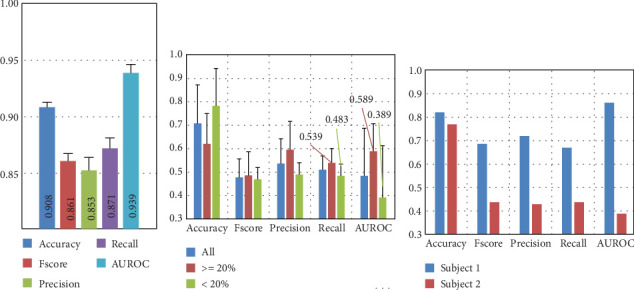
Evaluating the generalization of the machine learning algorithms for dream and dreamless classification. (a) Average performance of the models when leaving one subject out of the training set. (b) Average classification performance for the excluded subjects, considering all the subjects, those with ≥ 20% instances in the minority class, and those with < 20% instances in the minority class. (c) Example of best and worst performance when leaving one subject out of the training set. Note: AUROC, area under the receiver operating characteristic curve.

Figure 8(a) shows the average performance in the training sets with one subject excluded at a time. The results are similar to those of Figure 5, especially with CSP-based feature extraction and KNN, except for the precision and recall metrics.

Figure 8(b) shows the average performance on the excluded subjects' data. The results indicate a high standard deviation and accuracy exceeding 0.7, but performance for all other metrics appears to be random. To explore whether random performance is associated with class imbalance in the subject excluded from the training set, we also plot the results, including only subjects where the percentage of the class with fewer instances is *≥* 20, and also separating the subset of subjects if the percentage of the class with fewer instances is *<* 20. The results show that when considering at least a class distribution of 20% and 80%, the performance slightly increases in most metrics, but the standard deviation remains high.

Due to the high standard deviation, Figure 8(c) presents the performance for the subjects with the highest and lowest performance. These findings demonstrate that the ML model can classify EEG signals from excluded subjects as dream or dreamless, with some subjects achieving performance above 0.65 in all metrics.

### 3.4. Permutation-Based Channel Selection for EEG-Based Dream and Dreamless Classification

As demonstrated by testing on unseen data (i.e., leave-one-subject-out of the training set), the performance remains random at this stage of our research. Thus, this aspect is reserved for future work. Our current focus is on using a minimal number of EEG channels for dream and dreamless classification with data from all available subjects.

**Figure 9:**
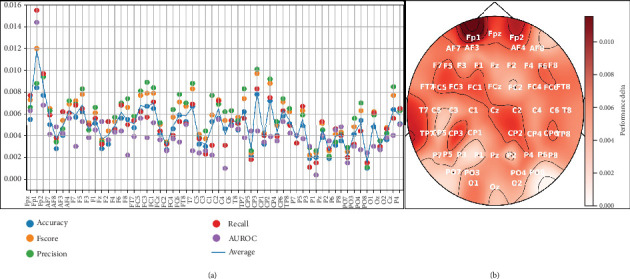
Permutation-based channel selection for dream and dreamless classification. (a) Channel importance per metric and the average importance among them. (b) Topographic map showing average performance from 10-fold cross-validation and metrics used. Note: AUROC, area under the receiver operating characteristic curve.

One limitation of our previous work was the use of only six EEG channels [27]. In this experiment, we applied a permutation-based channel selection method to identify the most relevant EEG channels for dream and dreamless classification. The average results obtained after 10-fold cross-validation are presented in Figure 9 and illustrate how much performance decreases when we remove a specific channel from the test set. For example, if instead of using the EEG signal from *Fpz*, we use random values, the *F*score decreases by 0.012 from the baseline performance (which is when using data from the *Fpz* channel), and the AUROC decreases by around 0.015. This highlights *Fpz* as a crucial EEG channel for dream and dreamless classification.

To provide a clear overview of the most relevant channels according to the permutation-based channel selection, Figure 9 presents a topographic map where the most important areas are shown in darker shades.

**Figure 10:**
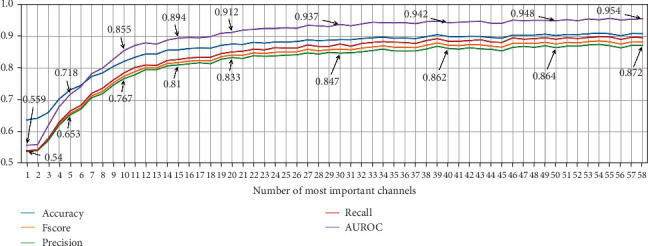
ML model performance for dream and dreamless classification using the *k* most important channels identified by the permutation-based channel selection method and presented in Figure 9. Note: AUROC, area under the receiver operating characteristic curve.

Based on the identified relevant channels, we conducted experiments using from 1 to 58 of the most important channels to observe performance trends across different metrics.  Figure 10 shows that the performance remains stable with more than 25 EEG channels, with only minor fluctuations compared to using all 58 channels.

### 3.5. NSGA-Based Channel Selection for Dream Versus Dreamless Classification

The findings of the previous experiment are relevant, as they align with those reported by the authors of the dataset. However, the performance loss according to the importance of the channels in Figure 9 is under 2% at worst, suggesting that similar performance could be achieved with different EEG channel combinations.

We used the NSGA-II algorithm to determine the optimal EEG channels to obtain the highest classification performance. NSGA-II has been shown to be robust when selecting the optimal EEG channels for different EEG-related tasks [27, 41]. For this, we defined six objectives to be optimized; Objectives 1–5 aim to maximize the five performance metrics, while Objective 6 minimizes the number of EEG channels used to create the ML models.

**Figure 11:**
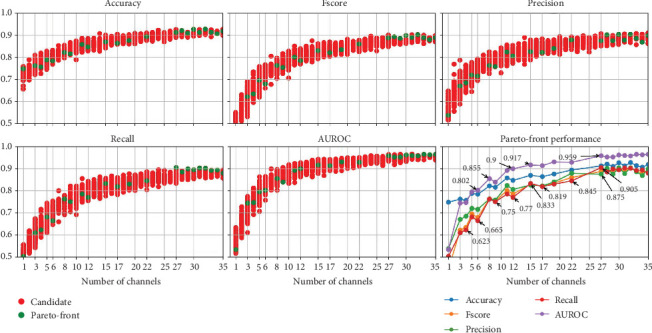
Performance evolution for dream and dreamless classification using the nondominated sorting genetic algorithm II (NSGA-II) for channel selection. Note: AUROC, area under the receiver operating characteristic curve.

Figure 11 presents the results of the NSGA-II optimization, with subplots for each optimized metric as the number of channels decreases and a subplot for performance on the Pareto front. Although the dataset includes 58 EEG channels, the results show that using at least 27 EEG channels yields low-performance variation and achieves the highest possible performance compared to the results presented in Figure 5. Thus, we present performance graphs for one to 35 EEG channels.

The results indicate that when using less than 10 EEG channels to create ML models, the performance starts to decrease drastically. This is also shown in the results of the permutation-based channel in Figure 10. However, the NSGA-based channel selection method achieves higher performance, with AUROC reaching 0.8 with five EEG channels compared to 0.718 with the permutation-based channel selection method.

We decided not to include the selected channels using the NSGA-based method because the results in the Pareto front come from different channel combinations, which may not consistently include the EEG channels deemed optimal by other combinations. For example, supposing that the “Fp1” channel is in the Pareto front when using only one channel, it does not mean that the results when using 3 EEG channels of the Pareto front include the “Fp1” channel. And a single topographic map showing the selected channels in the Pareto front covers the entire scalp, which is consistent with previous findings that argue that the differences between EEG signals with dream and dreamless content are global rather than localized [17].

### 3.6. Exploring the Impact of Random Combinations of EEG Channels for Dream and Dreamless Classification

**Figure 12:**
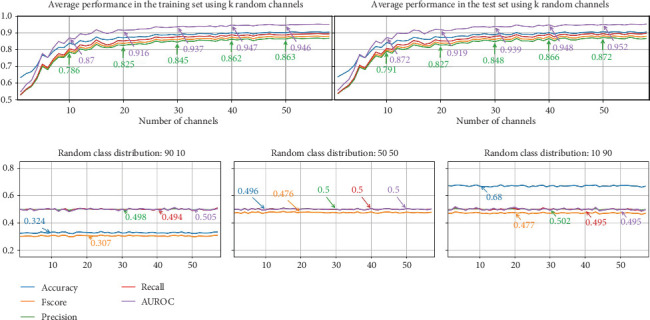
Performance of dream and dreamless classification with random channel sets. The figure shows the average performance evolution (average performance after 10-fold cross-validation) when using a single random electroencephalographic channel or up to all 58 electroencephalographic channels. The three lower subfigures present the performance evaluating the created models with *k* random channels but using random labels for the test sets. Note: AUROC, area under the receiver operating characteristic curve.

The upper subfigures of Figure 12 present the average results for the EEG-based dream versus dreamless classification using random channel combinations. Each experiment was repeated 10 times with randomly assigned channel combinations for each fold.

The results indicate that when using random combinations of channels, the highest performance is comparable to that achieved with permutation-based channel selection. These findings also suggest that NSGA-II can effectively identify channels with better performance and that other random channel combinations may not be able to match the performance of the optimal channel combination identified by NSGA-II.

The difference between the approaches for channel selection is more pronounced with fewer than 15 EEG channels. Using 10 random EEG channels yielded the following performance: accuracy: 0.842, *F*score: 0.798, precision: 0.791, recall: 0.807, and AUROC: 0.872.

The lower subfigures of Figure 12 include the results of a set of experiments in which we randomly assign labels to test sets 10 times (10-fold cross-validation) and then use models trained with *K* = 1 − 58 channels and with the correct labels to predict the classes in the test sets. First, we show the performance obtained with random labels to obtain a class distribution of *[90 10]*, then *[50 50]*, and *[10 90]*. In all three cases, the performance obtained is random, clearly indicating that the EEG signals from dream and dreamless reports are correctly associated. Balancing the dataset across any of the classes does not improve performance metrics, except accuracy (see the class distribution *[10 90]* from Figure 12). This simple exercise shows the importance of using different metrics for the evaluation of performance due to the unbalanced datasets that can be obtained from dream reports.

## 4. Discussion

This study contributes to ongoing scientific inquiry into the purpose and mechanisms of dreaming, focusing on the automatic detection of dream experiences through feature extraction and ML-based methods. We presented a comprehensive approach to automatic dream detection using EEG signals from a publicly available dataset that includes dream reports from the REM and NREM sleep stages [17].

We applied CSP and DWT for feature extraction, followed by various ML algorithms for classification, highlighting KNN as the top method. The preprocessing steps involved downsampling, re-referencing, and segmentation of EEG data, setting the stage for robust feature extraction. We implemented CSP-based feature extraction to capture class-specific spatial patterns and DWT-based feature extraction to represent the time-frequency characteristics of the EEG signals from dream and dreamless states.

In this study, we analyzed the impact of class imbalance, showing that performance remained relatively stable, suggesting that data balancing may not be too crucial but still relevant to increase performance in certain metrics. Our findings support the applicability of this approach to real-world dream datasets with unequal class sizes. The robustness of the models was demonstrated by low standard deviations during the 10-fold cross-validation, emphasizing the reliability and consistency of the proposed approach.

Our research showed promising results in automatic dream detection during REM and NREM sleep, particularly using CSP-based feature extraction with KNN in multiple combinations of EEG channels (of up to 58 channels). The classification performance to distinguish between dream and dreamless experiences exceeded 0.85 in many metrics.

We addressed the challenges associated with the computational cost and high dimensionality of the EEG data by exploring channel reduction and selection techniques. The permutation-based and NSGA-II channel selection methods were used to identify the most relevant EEG channels for the classification task and to select a set of channels that allow focus on the most important areas of the brain associated with dreams and dreamless experiences. The permutation-based channel selection method identified the most relevant channels, with a minimal performance decline for subsets containing more than 25 EEG channels. However, channel selection using NSGA-II outperformed permutation-based channel selection by achieving higher performance with fewer channels. The best combinations from the NSGA-II Pareto fronts suggest that as few as eight to 10 EEG channels can suffice, offering a foundation for lighter EEG systems for dream recognition.

The results of permutation-based channel selection are consistent with previous research, which found increased frontal/prefrontal cortex activity during REM sleep when subjects reported dream experiences, suggesting that the frontal region may play a key role in dream recall [6]. Further analysis is needed to fully understand the role of the frontal/prefrontal area in dream classification, as the EEG channels Fp1 and Fp2 can be affected by blinks and eye movements, particularly during REM sleep and sleep onset [60–62]. This could be explored by developing ML models that use data solely from REM or NREM dream reports, allowing a comparison of channel importance in each case. Since NSGA-II selected channel subsets from different brain areas, this may be related to the inclusion of both REM and NREM dream reports in a single model for dream classification.

Taking advantage of the same dataset and other public datasets [6, 17], we plan to investigate whether the REM dream reports remain localized in the frontal/prefrontal areas or if there are differences, as the authors of [6] also found differences in the posterior cortical area during NREM and REM, reporting that a decrease in low frequencies is related to the report of dream experiences, and an increase in low frequencies is associated with no experiences, with results analyzed at the source level.

By trying to associate dream experiences with specific cortical regions, the research advances our knowledge of the neuroanatomical correlates of dreaming during different sleep stages, and it aligns with broader trends in neuroscience that recognize individual variability in brain function [6, 7, 17, 22, 25–28, 32–36].

Furthermore, we investigated the use of random combinations of EEG channels to discern the impact of channel selection strategies. Surprisingly, random sets of channels exhibited a performance comparable to permutation-based channel selection, underscoring the need for caution when interpreting results solely based on classification metrics and a single method for channel selection. The experiments showed that using one to two EEG channels, whether with NSGA-II, permutation-based channel selection, or random sets of channels, resulted in comparable performance.

Experiments that involved the exclusion of one subject at a time during model training demonstrated challenges in generalizing the models to unseen subjects, highlighting potential limitations in model transferability. Our results suggest that future research should prioritize larger, more diverse datasets to enhance the generalizability and transferability of trained models, which are currently limited by the number of dream reports and subjects [17, 24, 28, 29].

One limitation of the dataset studied in this work is the collection of *sleep onset* dream reports that were collected very close to each other, which could result in subjects being awake or reporting the same or similar dream experiences [17]. Given the limited dataset, only 73 s of EEG data related to dream experiences were available, later segmented into 2-s segments. While this may impact model performance and further analysis of this point is needed, using short segments helps increase instances for training ML models, as shown in our previous work [27].

The authors who collected the dataset used in our study argue that they tried to maximize the number of dream reports by collecting them after awakening during the sleep onset period because dream recall is high and subjects can quickly return to sleep [17]. However, as shown in Figure 1, their publication and documentation in the DREAM project ^1^ show that most dreamless experiences occur during sleep onset. Our research suggests that detecting when a subject is dreaming is easier during REM sleep [27]; however, we have also shown that the classification performance is similar using only NREM/REM data [30, 31].

Our current study shows that balancing the dataset does not improve classification performance compared to using unbalanced datasets. However, future research should consider larger, balanced datasets to assess performance on unseen data (epochs and subjects). Our future work will explore factors such as using balanced datasets (using REM, NREM, or both in the same ML/DL models), using the serial awakening paradigm, and when and at what time subjects reported most dream experiences, among other considerations.

As discussed previously [27], future research should control critical experimental factors such as number of channels, sample rate, age, sex, and others. Controlling these factors can improve performance. For example, studies show that clustering algorithms often group instances by characteristics unrelated to dream content [7]. Another study found that men's dreams are more often linked to physical aggression and sexual content than women's [4].

Automatic dream detection, although in its early stages, has the potential to transform fields, including sleep research, clinical diagnosis, personalized therapies, and even creative expression. Automatic dream detection can significantly accelerate research by providing objective and continuous data on dream experiences, helping to understand their role in memory consolidation, emotional processing, and other brain functions.

The subjective nature of dreams, along with individual differences in their experience and expression, presents major challenges for ML/DL algorithms trained on limited data. Inconsistencies in findings on brain activity linked to dreaming further complicate the development of robust and universal detection methods [6, 17, 32–36]. However, advances in feature extraction, ML/DL techniques, and data availability offer promising opportunities for the development of more accurate dream detection models [17, 24, 28, 29].

As the field progresses, the integration of real-time monitoring and decoding of dream experiences using ML/DL holds exciting prospects. Developing low-density wearable devices capable of real-time dream detection could not only deepen our understanding of the nature of dreams but also pave the way for personalized therapeutic interventions for sleep disorders and mental health conditions. The journey to unravel the secrets of sleep and consciousness continues, with automatic dream detection poised to play a pivotal role in unlocking the mysteries of the nocturnal mind.

## Data Availability

The dataset analyzed for this study can be found in the DREAM project [17, 28] (doi:10.26180/22133105).

## Endnotes

^1^The dataset from the DREAM project used in our study is accessible through its DOI (10.6084/m9.figshare.22226692).

## References

[1] Calkins M. W. (1893). Statistics of dreams. *The American Journal of Psychology*.

[2] Hobson J. A., McCarley R. W. (1977). The brain as a dream state generator: an activation-synthesis hypothesis of the dream process. *The American Journal of Psychiatry*.

[3] Hobson J. A. (2009). REM sleep and dreaming: towards a theory of protoconsciousness. *Nature Reviews Neuroscience*.

[4] Winkelman J. W., Plante D. T. (2011). *Foundations of Psychiatric Sleep Medicine*.

[5] Schredl M. (2010). *Dream content analysis: basic principles*.

[6] Siclari F., Baird B., Perogamvros L. (2017). The neural correlates of dreaming. *Nature Neuroscience*.

[7] Wong W., Noreika V., Móró L. (2020). The Dream Catcher experiment: blinded analyses failed to detect markers of dreaming consciousness in EEG spectral power. *Neuroscience of Consciousness*.

[8] Scarpelli S., Bartolacci C., D’Atri A., Gorgoni M., De Gennaro L. (2019). The functional role of dreaming in emotional processes. *Frontiers in Psychology*.

[9] Moffitt A., Kramer M., Hoffmann R. (1993). *The Functions of Dreaming*.

[10] Staunton H. (2001). The function of dreaming. *Reviews in the Neurosciences*.

[11] Okuma T. (1992). On the psychophysiology of dreaming: a sensory image——free association hypothesis of the dream process. *Psychiatry and Clinical Neurosciences*.

[12] Horowitz A. H., Esfahany K., Gálvez T. V., Maes P., Stickgold R. (2023). Targeted dream incubation at sleep onset increases post-sleep creative performance. *Scientific Reports*.

[13] Nielsen T. A. (2000). A review of mentation in REM and NREM sleep:“covert” REM sleep as a possible reconciliation of two opposing models. *Behavioral and Brain Sciences*.

[14] Hobson J. A., Pace-Schott E. F., Stickgold R. (2000). Dreaming and the brain: toward a cognitive neuroscience of conscious states. *Behavioral and Brain Sciences*.

[15] Noreika V., Valli K., Lahtela H., Revonsuo A. (2009). Early-night serial awakenings as a new paradigm for studies on NREM dreaming. *International Journal of Psychophysiology*.

[16] Nir Y., Tononi G. (2010). Dreaming and the brain: from phenomenology to neurophysiology. *Trends in Cognitive Sciences*.

[17] Zhang J., Wamsley E. J. (2019). EEG predictors of dreaming outside of REM sleep. *Psychophysiology*.

[18] Hoel E. (2021). The overfitted brain: dreams evolved to assist generalization. *Patterns*.

[19] Cipolli C., Ferrara M., De Gennaro L., Plazzi G. (2017). Beyond the neuropsychology of dreaming: insights into the neural basis of dreaming with new techniques of sleep recording and analysis. *Sleep Medicine Reviews*.

[20] Siclari F., LaRocque J. J., Postle B. R., Tononi G. (2013). Assessing sleep consciousness within subjects using a serial awakening paradigm. *Frontiers in Psychology*.

[21] Idir Y., Oudiette D., Arnulf I. (2022). Sleepwalking, sleep terrors, sexsomnia and other disorders of arousal: the old and the new. *Journal of Sleep Research*.

[22] Sikka P., Revonsuo A., Noreika V., Valli K. (2019). EEG frontal alpha asymmetry and dream affect: alpha oscillations over the right frontal cortex during rem sleep and presleep wakefulness predict anger in REM sleep dreams. *Journal of Neuroscience*.

[23] Yousef G., Florence S. M. Dream classification based on beta waves in EEG signals.

[24] Zheng L., Zhou D., Zhang M. (2022). DEED: A Multimodel Dataset For Dream Emotion Classification.

[25] Horikawa T., Tamaki M., Miyawaki Y., Kamitani Y. (2013). Neural decoding of visual imagery during sleep. *Science*.

[26] Horikawa T., Kamitani Y. (2017). Hierarchical neural representation of dreamed objects revealed by brain decoding with deep neural network features. *Frontiers in Computational Neuroscience*.

[27] Moctezuma L. A., Ipanaque F., Molinas M., Abe T. Dream emotions identified without awakenings by machine and deep learning from electroencephalographic signals in REM sleep.

[28] Wong W., Andrade K. C., Andrillon T. (2023). *DREAM: A Dream EEG and Mentation Database*.

[29] Liu W., Zhang Y., Ma P. (2022). *DEED: A Dataset for Dream-related Emotion Research*.

[30] Torvestad A., Packiyanathan M., Moctezuma L. A., Molinas M. Unveiling dreams: moving towards automatic dream decoding via qualitative EEG analysis and machine learning.

[31] Packiyanathan M., Torvestad A., Moctezuma L. A., Molinas M. Exploring high-and low-density electroencephalography for a dream decoding brain-computer interface.

[32] Esposito M. J., Nielsen T. A., Paquette T. (2004). Reduced Alpha power associated with the recall of mentation from stage 2 and stage REM sleep. *Psychophysiology*.

[33] Takeuchi T., Ogilvie R. D., Murphy T. I., Ferrelli A. V. (2003). EEG activities during elicited sleep onset REM and NREM periods reflect different mechanisms of dream generation. *Clinical Neurophysiology*.

[34] Chellappa S. L., Muench M., Knoblauch V., Cajochen C. (2012). Age effects on spectral electroencephalogram activity prior to dream recall. *Journal of Sleep Research*.

[35] Nielsen T., Carr M., Blanchette-Carrière C. (2017). NREM sleep spindles are associated with dream recall. *Sleep Spindles & Cortical Up States*.

[36] Scarpelli S., D’Atri A., Mangiaruga A. (2017). Predicting dream recall: EEG activation during NREM sleep or shared mechanisms with wakefulness?. *Brain Topography*.

[37] Cataldi J., Stephan A. M., Haba-Rubio J., Siclari F. (2024). Shared EEG correlates between non-REM parasomnia experiences and dreams. *Nature Communications*.

[38] Pedregosa F., Varoquaux G., Gramfort A. (2011). Scikit-learn: machine learning in python. *The Journal of Machine Learning Research*.

[39] Mallat S. G. (1989). A theory for multiresolution signal decomposition: the wavelet representation. *IEEE Transactions on Pattern Analysis and Machine Intelligence*.

[40] Moctezuma L. A., Abe T., Molinas M. (2022). Two-dimensional CNN-based distinction of human emotions from EEG channels selected by multi-objective evolutionary algorithm. *Scientific Reports*.

[41] Moctezuma L. A. (2021). *Towards Universal EEG Systems With Minimum Channel Count Based on Machine Learning and Computational Intelligence, [Ph.D. thesis]*.

[42] Duan R.-N., Zhu J.-Y., Lu B.-L. Differential entropy feature for EEG-based emotion classification.

[43] Singhal Y., Jain A., Batra S., Varshney Y., Rathi M. Review of bagging and boosting classification performance on unbalanced binary classification.

[44] Bentéjac C., Csörgő A., Martínez-Muñoz G. (2021). A comparative analysis of gradient boosting algorithms. *Artificial Intelligence Review*.

[45] Brown I., Mues C. (2012). An experimental comparison of classification algorithms for imbalanced credit scoring data sets. *Expert Systems With Applications*.

[46] Tanha J., Abdi Y., Samadi N., Razzaghi N., Asadpour M. (2020). Boosting methods for multi-class imbalanced data classification: an experimental review. *Journal of Big Data*.

[47] Sen P. C., Hajra M., Ghosh M., Mandal J., Bhattacharya D. (2020). Supervised classification algorithms in machine learning: a survey and review. *Emerging Technology in Modelling and Graphics. Advances in Intelligent Systems and Computing*.

[48] Wright R. E., Grimm L. G., Yarnold P. R. (1995). Logistic regression. *Reading and Understanding Multivariate Statistics*.

[49] Menard S. (2002). Applied logistic regression analysis. *Quantitative Applications in the Social Sciences 106*.

[50] Joachims T. (1999). Making large-scale support vector machine learning practical. *Advances in Kernel Methods: Support Vector Learning*.

[51] Peterson L. E. (2009). K-nearest neighbor. *Scholarpedia*.

[52] Taunk K., De S., Verma S., Swetapadma A. A brief review of nearest neighbor algorithm for learning and classification.

[53] LeCun Y., Bengio Y., Hinton G. (2015). Deep learning. *nature*.

[54] Alotaiby T., El-Samie F. E. A., Alshebeili S. A., Ahmad I. (2015). A review of channel selection algorithms for EEG signal processing. *EURASIP Journal on Advances in Signal Processing*.

[55] Moctezuma L. A., Suzuki Y., Furuki J., Molinas M., Abe T. (2024). GRU-powered sleep stage classification with permutation-based EEG channel selection. *Scientific Reports*.

[56] Moctezuma L. A., Suzuki Y., Furuki J., Molinas M., Abe T. Enhancing sleep stage classification with 2-class stratification and permutation-based channel selection.

[57] Deb K. (2011). Multi-objective optimisation using evolutionary algorithms: an introduction. *Multi-Objective Evolutionary Optimisation for Product Design and Manufacturing*.

[58] Srinivas N., Deb K. (1994). Muiltiobjective optimization using nondominated sorting in genetic algorithms. *Evolutionary Computation*.

[59] Deb K., Pratap A., Agarwal S., Meyarivan T. (2002). A fast and elitist multiobjective genetic algorithm: NSGA-II. *IEEE Transactions on Evolutionary Computation*.

[60] Dement W., Kleitman N. (1957). The relation of eye movements during sleep to dream activity: an objective method for the study of dreaming. *Journal of Experimental Psychology*.

[61] Rowley J. T., Stickgold R., Hobson J. A. (1998). Eyelid movements and mental activity at sleep onset. *Consciousness and Cognition*.

[62] Maranci J.-B., Nigam M., Masset L. (2022). Eye movement patterns correlate with overt emotional behaviours in rapid eye movement sleep. *Scientific Reports*.

